# Mycotoxin production in different varieties of *Dactylis glomerata* L. silage in response to biological and chemical additives

**DOI:** 10.1371/journal.pone.0309662

**Published:** 2024-08-29

**Authors:** Jhonny E. Alba-Mejía, Gloria Domínguez-Rodríguez, Tomáš Středa, Hana Středová, Lea Lojková, Pavel Horký, Sylvie Skaličková, Jiří Skládanka

**Affiliations:** 1 Department of Crop Science, Breeding and Plant Protection, Faculty of AgriSciences, Mendel University in Brno, Brno, Czech Republic; 2 Departamento de Química Analítica, Química Física e Ingeniería Química, Facultad de Ciencias, Universidad de Alcalá, Alcalá de Henares, Madrid, Spain; 3 Department of Applied and Landscape Ecology, Faculty of AgriSciences, Mendel University in Brno, Brno, Czech Republic; 4 Department of Animal Nutrition and Forage Production, Faculty of AgriSciences, Mendel University in Brno, Brno, Czech Republic; Zagazig University Faculty of Agriculture, EGYPT

## Abstract

Silage has been identified as a source of different microbial toxins, that may impair farm animal health and productivity as human health can also be compromised. In this sense, the aim of this study was to determine the impact of silage additives on the concentrations of deoxynivalenol (DON) and zearalenone (ZEN) mycotoxins and, eventually, to evaluate the hygienic quality of orchardgrass (*Dactylis glomerata* L.) silage based on the concentration of them compared to control silage. This study evaluated the influence of biological and chemical additives used in six different varieties of orchardgrass silage on DON and ZEN mycotoxin contents for the first time. The content of both fusariotoxins (DON and ZEN) in fresh matter and grass silage were below the threshold stipulated by the European Commission. The concentration of DON ranges from ~21.86 to 37.26 ng/kg, ~10.21 to 15 ng/kg, ~20.72 to 29.14 ng/kg; and ZEN range from ~3.42 to 7.87 ng/kg, ~3.85 to 8.62 ng/kg and ~2.15 to 5.08 ng/kg, in control, biological and chemical silages, respectively. In general, the biological additive was more efficient for preventing DON contamination, whereas the chemical additive was more efficient for preventing ZEN contamination in grass silage. In summary, the results obtained in this work demonstrate that biological and chemical additives can inhibit fungal growth and mycotoxin production on *Dactylis glomerata* L. silage and whose use could prevent animal and human diseases.

## Introduction

Mycotoxins are toxic secondary metabolites synthetized by certain filamentous fungi species that typically grow during pre-harvest (e.g., *Alternaria* spp. and *Fusarium* spp.), post-harvest (e.g., *Penicillium* spp.) or at both times (e.g., *Aspergillus* spp.) under suitable environmental conditions [1]. Various factors such as climate, storage conditions, and agricultural practice are known to influence the incidence of mycotoxins and they may occur in a wide range of animal feeds, including concentrates, forage grass, and silage [2].

Particularly, during ensiled periods, silage is vulnerable to contamination by mycotoxigenic fungi being a risk for dairy cows which are fed mainly by silage, thereby affecting animal performance [3, 4]. Deoxynivalenol (DON) as well as zearalenone (ZEN) were the most frequently mycotoxins, present in grass and maize silages from Austria. Likewise, DON and ZEN were found in maize silage from Poland, while other mycotoxins such as beauvericin have been found in grass silages as well as roquefortine C in whole-crop cereal silages from Finland.

Of the more than 500 know mycotoxins, 30 are considered a risk for human or animal health [5]. However, European Union legislation regulates only five mycotoxins which are aflatoxins, ochratoxins, fumonisins, DON and ZEN [6]. Among them, the European Food Safety Authority (EFSA) ranks DON and ZEN as undesirable substances of the agri-food chain. DON causes undesirable effects for dairy cows, including loss of appetite, reduced rumination activity, and feed intake, as well as the upregulation of pro-inflammatory cytokines and immuno-suppression [7]. On the other hand, ZEN is a potent nonsteroidal, genotoxic estrogenic mycotoxin that has been reported to cause abortion and hyperestrogenism, and reduced fertility in livestock [8]. For these reasons, the European Commission limited the DON concentrations to 5 mg/kg for cattle, horses and poultry feed, and 2 mg/kg applies for calves (<4 months of age), lambs and kids; while the ZEN concentration was limited to 0.5 mg/kg for calves, dairy cattle, sheep (including lamb) and goats (including kids); related complete feedstuffs with 88% dry matter (DM) content for ruminants [9–11].

In order to prevent contamination by mycotoxins in animal feeds minimizing nutrient and energy losses, biological and chemical silage additives have been employed [12]. Different lactic acid bacteria (LAB) have been used as biological additives to enhance silage fermentation or aerobic stability in silages, thereby delaying the development of yeast and spoilage molds [13]. The most used biological silage inoculants during the ensiled process are based on the fermentative pathway, that includes homofermentative LAB (e.g., *Pediococcus damnosus* and *Lactobacillus ruminis*), facultatively heterofermentative LAB (e.g., *L*. *plantarum*, *L*. *pentosus*, *P*. *acidilactici*, *P*. *pentosaceus* and *Enterococcus faecium*), and obligately heterofermentative LAB such as *L*. *buchneri*, *L*. *brevis* [14] and *L*. *parafarraginis* [15].

It has been documented that biological additives can degrade or immobilize aflatoxins during ensiling by binding to their surface [16, 17], thereby contributing to the improved hygienic silage safety. However, responses to biological silage additives could be influenced by several factors, including the type of forage, the type of additive, the application rate of additives, and other ensilage management processes during pre- and post-ensiling practices [18], as well as by the climatological conditions, among other things [19].

On the other hand, acids and their salts are used as chemical additives to alter the pH of silage, creating an unsuitable environment for fungal growth. Chemical additives traditionally fall into two groups: i) formic acid, which causes direct acidification, suppressing clostridia and other undesirable spoilage bacteria, and improving protein preservation during ensiling, and ii) sorbic, benzoic, propionic, and acetic acids, which improve silage aerobic stability through the direct inhibition of yeasts and molds [13]. Current research has focused on combinations of these acids to improve silage hygienic safety. Besides, recent studies conducted in Slovakia with maize silage and Finland with grass silage showed that chemical additives, positively reduce ZEN concentration [20, 21].

To our knowledge the studies about the influence of different biological and chemical additives on grass silage is very limited. In fact, the influence of the application of these additives on fermentation parameters in different varieties of grass silage was previously evaluated by Alba-Mejía et al. [22]. In their study, the authors observed that the orchardgrass varieties treated with biological additives improved the quality of the fermentation process in silage. However, the influence of biological and chemical additives on DON and ZEN contamination in *Dactylis glomerata* L. has not been studied. For this reason, the results obtained by Alba-Mejía et al. [22] have been taken into account.

The aim of this study was to determine the effects of biological and chemical additives on the DON and ZEN levels of grass silage according to different varieties of Dactylis glomerata L. by comparison with untreated silage. Besides, the hygienic quality of silage based on the current levels of both fusariotoxins in orchardgrass silage and fresh matter was evaluated.

## Material and methods

The study was conducted according to the guidelines of the Institutional Review Board of Expert Commission of Mendel University in Brno (protocol code 16OZ27083/2014-17214).

### Fresh raw materials

Six orchardgrass varieties (*Dactylis glomerata* L.) belonging to Greenly, Sw-Luxor, Otello, Husar, Bepro and Dana varieties from France, Sweden, Italy, Germany, Poland, and Czech Republic, respectively, were used in this research. The trial was established in Czech Republic at the Research Station of Fodder Crops in Vatín (49°31′N, 15°58′E, 560 m.a.s.l.). The research site presented total precipitations and the average annual air temperature of 632, 658, and 705 mm and 7.4, 6.8 and 7.3°C in 2011, 2012, and 2013, respectively. The experimental design consisted of a plot with a soil type of Cambisol with a sandy-loam texture with a dimension of 1.5 m × 10 m (three replicates). For harvesting, a self-propelled mowing machine with a mowing width of 1.25 m was used. The harvested area of individual plots was 12.5 m^2^, with a remaining stubble height of 7 cm where 20 kg/ha dose of seeds was applied during sowing. The fertilization consisted of a 60 kg/ha N dose per year in urea form. The second growth of the whole plant was harvested at the heading phase. The forage was wilted on the plot for 14 h to reduce the water content after harvest. Afterward, the forage samples (10 kg per treatment) were taken to the laboratory and chopped with a conventional electric forage cutter to a particle length of 40–60 mm.

### Silage preparation

Representative forage samples (6 kg) were placed in mini-silos of polyvinyl chloride (PVC) and compacted to a pressure of 600 kg/m^-3^. The mini silos (three repetitions per treatment) were sealed with a lid and stored at room temperature (28°C) for three months. In order to evaluate the effectivity of additives in the orchardgrass silage, the following treatments were applied to the forage:

- Control silage without inoculants (only with deionized water).
- Biological treatment with a mixture of *Lactobacillus plantarum* (DSMZ 16568) in a concentration of 5 × 10^10^ colony-forming unit (CFU)/g, *Lactobacillus buchneri* (DSMZ 22501/CCM 1819, DSZM: German collection of microorganisms and cell cultures; CCM: Czech collection of microorganisms) in a concentration of 1.25 × 10^10^ CFU/g, and *Enterococcus faecium* (DSMZ 22502) in a concentration of 6.25 × 10^10^ CFU/g (manufacturer CHR. HANSEN A/S, Denmark). The total bacterial concentration in the product was 12.5 x 10^10^ CFU/g and the recommended dose is 250 000 CFU/g of fresh forage.
- Chemical treatment with a mixture of formic acid (43% w/w), propionic acid (10% w/w), benzoic acid (2% w/w), ammonium formate (30% w/w), E150d-sulfite ammonia caramel and water (15% w/w) (manufacturer BIOFERM CZ, spol. s r.o., Czech Republic).

The microbial inoculants (2 g/ton) were diluted in sterilized water and applied using a hand sprayer at a rate of 4 L/ton and the chemical additives were applied at a rate of 4 L/ton in both cases by spraying uniformly onto the forage grass, which was constantly hand mixed. The same amount (4 L/ton) of deionized water was added to the control treatment. At the end of the ensiling period (three months), the silos were opened, and samples were taken for analysis. Dry matter was analyzed after drying the biomass at 103°C. Meanwhile, the pre-drying silage samples was performed in a specific drying oven at 60°C for 48 h. Subsequently, the forage samples were ground in a mill and then filtered through a 1 mm sieve. The ground pre- and post-ensiled grass samples were stored under darkness at room temperature of 28°C until their analysis.

The content of both mycotoxins (DON and ZEN) in the fresh matter is presented in **Table 1**, while the levels of the ensiled grass are found in **Tables 2** and **3**.

**Table 1:** Concentration of DON and ZEN (ng/kg DM) in fresh matter.

Varieties	DON		ZEN	
	*2012*	*2013*	*2012*	*2013*
*Greenly*	32.40	38.14	0.49	2.15
*Sw-Luxor*	32.29	27.35	0.15	1.04
*Otello*	28.48	22.38	0.52	1.50
*Husar*	24.77	25.10	0.27	2.45
*Bepro*	23.10	22.53	0.05	1.77
*Dana*	36.84	22.91	0.00	1.33

**Table 2:** Concentration of DON in orchardgrass silage.

Varieties	DON (ng/kg DM)			
	*CS*	*BSA*	*CSA*	P value
*Greenly*	22.26 ± 2.30 ^**B**^_**ab**_	10.21 ± 2.79 ^**A**^_**b**_	28.90 ± 5.98 ^**AB**^_**a**_	**0.0274**
*Sw-Luxor*	28.19 ± 3.02 ^**B**^_**a**_	12.65 ± 1.97 ^**A**^_**b**_	28.52 ± 3.94 ^**AB**^_**a**_	**0.0080**
*Otello*	27.53 ± 5.63 ^**B**^_**a**_	13.55 ± 1.94 ^**A**^_**b**_	26.58 ± 2.63 ^**AB**^_**a**_	**0.0483**
*Husar*	26.58 ± 3.35 ^**B**^_**a**_	14.19 ± 2.94 ^**A**^_**b**_	29.14 ± 4.33 ^**A**^_**a**_	**0.0353**
*Bepro*	21.86 ± 2.65 ^**B**^_**a**_	15.00 ± 1.82 ^**A**^_**a**_	24.47 ± 5.54 ^**AB**^_**a**_	**0.2277**
*Dana*	37.26 ± 2.73 ^**A**^_**a**_	13.98 ± 4.05 ^**A**^_**b**_	20.72 ± 4.48 ^**B**^_**b**_	**0.0055**
P value	**0.0125**	**0.7038**	**0.2711**	
**Year**				
*2012*	23.87 ± 2.51 ^**B**^_**a**_	10.92 ± 1.54 ^**B**^_**b**_	19.62 ± 1.41 ^**B**^_**a**_	**<0.0001**
*2013*	30.69 ± 1.68 ^**A**^_**a**_	15.61 ± 1.02 ^**A**^_**b**_	33.15 ± 1.71 ^**A**^_**a**_	**<0.0001**
P value	**0.0067**	**0.0217**	**<0.0001**	

CS: control silage; BSA: biological silage additives; CSA: chemical silage additives.

^AB^Superscript letters indicate significant differences among silage varieties treated with and without additives; and years in the same column (*p* < 0.05) determined by Fisher´s test.

^ab^Subscript letters indicate significant differences among treatments employed in the same silage varieties; and years in the same row (*p* < 0.05) determined by Fisher´s test.

**Table 3:** Concentration of ZEN in orchardgrass silage.

Varieties	ZEN (ng/kg DM)			
	*CS*	*BSA*	*CSA*	P value
*Greenly*	6.44 ± 0.68 ^**AB**^_**a**_	6.26 ± 2.36 ^**AB**^_**a**_	3.58 ± 1.36 ^**AB**^_**a**_	**0.4131**
*Sw-Luxor*	3.42 ± 1.30 ^**C**^_**a**_	3.85 ± 0.82 ^**B**^_**a**_	2.15 ± 0.83 ^**B**^_**a**_	**0.4909**
*Otello*	4.71 ± 0.96 ^**BC**^_**ab**_	6.38 ± 0.87 ^**AB**^_**a**_	2.88 ± 0.51 ^**AB**^_**b**_	**0.0386**
*Husar*	7.22 ± 0.66 ^**A**^_**a**_	8.62 ± 0.70 ^**A**^_**a**_	5.08 ± 0.63 ^**A**^_**b**_	**0.0134**
*Bepro*	7.87 ± 1.37 ^**A**^_**a**_	7.01 ± 1.03 ^**A**^_**a**_	5.02 ± 1.06 ^**A**^_**a**_	**0.2582**
*Dana*	4.43 ± 2.05 ^**BC**^_**a**_	6.20 ± 0.97 ^**AB**^_**a**_	5.06 ± 1.28 ^**A**^_**a**_	**0.7113**
P value	**0.0117**	**0.0537**	**0.0719**	
**Year**				
*2012*	6.16 ± 0.42 ^**A**^_**a**_	6.11 ± 0.63 ^**A**^_**a**_	3.94 ± 0.73 ^**A**^_**b**_	**0.0214**
*2013*	5.21 ± 1.06 ^**A**^_**ab**_	6.66 ± 0.90 ^**A**^_**a**_	3.98 ± 0.50 ^**A**^_**b**_	**0.1002**
P value	**0.1703**	**0.4627**	**0.9463**	

CS: control silage; BSA: biological silage additives; CSA: chemical silage additives.

^AB^Superscript letters indicate significant differences among silage varieties treated with and without additives; and years in the same column (*p* < 0.05) determined by Fisher´s test.

^ab^Subscript letters indicate significant differences among treatments employed in the same silage varieties; and years in the same row (*p* < 0.05) determined by Fisher´s test.

### Mycotoxins isolation and identification

Green fodder samples and silages were dried at 60°C, ground to a particle size of <1 mm, then analyzed for the content of mycotoxins, such as deoxynivalenol (DON) and zearalenone (ZEN), using the enzyme-linked immunosorbent assay (ELISA, Noack ČR, spol. s r. o., Czech Republic) according to Skládanka et al. [23].

Several analytical methods are available on the market for the detection and quantification of mycotoxins in feeds and foods. In particular, ELISA (enzyme-linked immunosorbent assay) is one of the most employed immunochemical techniques for the detection and quantification of mycotoxins due to their excellent specificity, simplicity, rapidity, and low cost [24–27]. Besides, ELISA is a robust method because provides high throughput by allowing the simultaneous testing of tens of samples and enables the detection of mycotoxins with high sensitivity and accuracy [28, 29].

### Statistical analyses

The software Statistica 14 (StatSoft CR s.r.o., Czech Republic) was used to perform the statistical analysis. The Shapiro–Wilk test (*p* < 0.05) was performed to check the normal distribution of the data. Analysis of variance one way (for fresh matter) and factorial (for silage) (ANOVA) by Fisher´s exact test was used to discriminate on the least significant difference LSD (*p* < 0.05) to compare differences of DON and ZEN concentrations among orchardgrass varieties treated with additives as well as control orchardgrass with a 95% confidence level. The results are expressed as the mean and standard error of mean (for tables) and mean and median (for figures).

A multivariate statistical analysis was performed using SIMCA 14.0 software (Umetrics, Sweden). The total DON and ZEN concentrations determined by ELISA after the biological and chemical additive treatments were used as variables. An unsupervised multivariate principal component analysis (PCA) was used to represent the statistical models as score plots.

## Results

A linear model was used to evaluate the relationship between the content of both mycotoxins (DON and ZEN) in the pre-ensiled (fresh matter) and post-ensiled grass (control variant). As a result, no clear relationship was identified between mycotoxins content in the fresh matter (**Table 1**) and ensiled grass (**Tables 2 and 3**). In the case of DON, the concentration of mycotoxins in ensiled grass increased with increasing mycotoxin levels in fresh matter (R2 = 0.555; not significant) in 2012. While in 2013, the levels of DON in ensiled grass decreased with increasing fresh matter (R2 = 0.379; not significant). Moreover, an opposite trend was found in 2012 for ZEN, where the content of this mycotoxin decreased in ensiled grass with increasing content in fresh matter (R2 = 0.448; not significant). While in 2013, ZEN content increased in ensiled grass with increasing content in fresh matter (R2 = 0.584; not significant). These results may indicate a mutual negative interaction between DON and ZEN producers.

Greenly, Sw-Luxor, Otello, Husar, Bepro, and Dana varieties of orchardgrass were treated with biological and chemical additives and compared with their corresponding untreated orchardgrass in terms of DON and ZEN contents.

As observed in **Table 2**, the untreated Dana variety was the most susceptible to contamination by DON, due to show the highest (*p* < 0.05) DON concentration compared with the rest of orchardgrass varieties. However, when the Dana variety was treated with biological additives, its DON concentration decreased obtaining a similar DON concentration to the rest of orchardgrass varieties biologically treated. In fact, the concentration of DON was reduced by half (*p* < 0.05) in all varieties when the biological additive was applied, except for the Bepro variety, where the reduction was moderate. Besides, Bepro is the only variety that did not show any significant differences (*p* > 0.05) among untreated and treated silage groups. In general, biological treatment was more efficient (*p* < 0.05) in preventing DON contamination than chemical additives. Furthermore, the concentration of DON was higher (*p* < 0.05) in 2013 compared with 2012 across all silage groups. In 2012 and 2013, the biological silage group presented lower (*p* < 0.05) concentrations of DON compared with the control and chemical silage groups.

On the other hand, Bepro along with Husar were the most susceptible varieties to contamination by ZEN (*p* < 0.05) due to these varieties presented the highest ZEN concentration in untreated silage (see **Table 3**). Differences in ZEN concentration among treated and untreated silage were not significant (*p* > 0.05), except for Otello and Husar varieties (*p* < 0.05). In fact, higher prevention of ZEN contamination for both varieties was observed when the chemical additive was used. Furthermore, in 2012, the concentration ZEN was lower (*p* < 0.05) in the chemical silage group compared to the control and biological silage groups; while in 2013, the statistical difference (*p* < 0.05) was observed only with the biological silage group.

**Fig 1:**
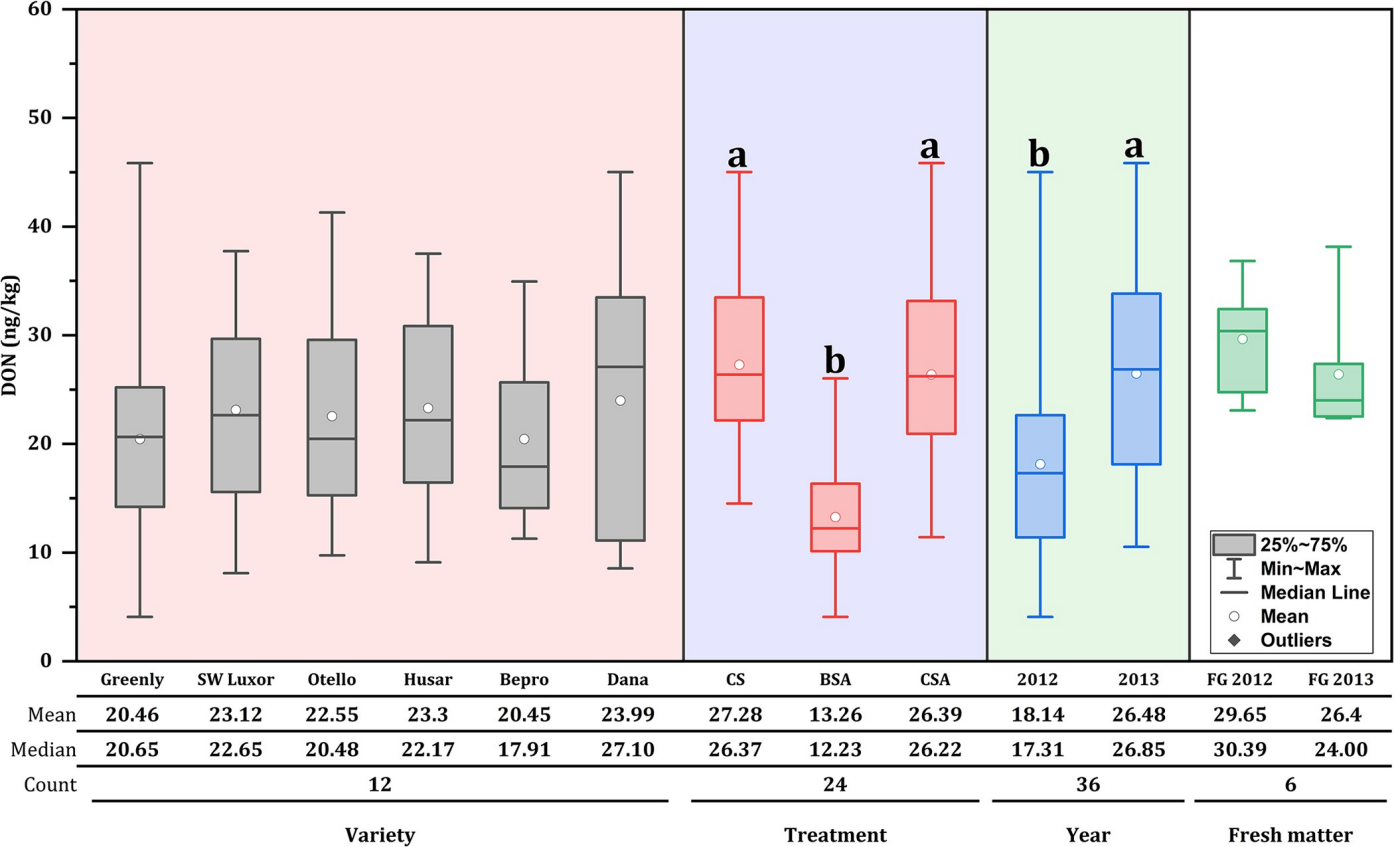
DON concentration (ng/kg DM) in six ensiled orchardgrass varieties and fresh matter. CS: Control silage; BSA: Biological silage additives; CSA: Chemical silage additives; FG: Fresh grass.

**Fig 2:**
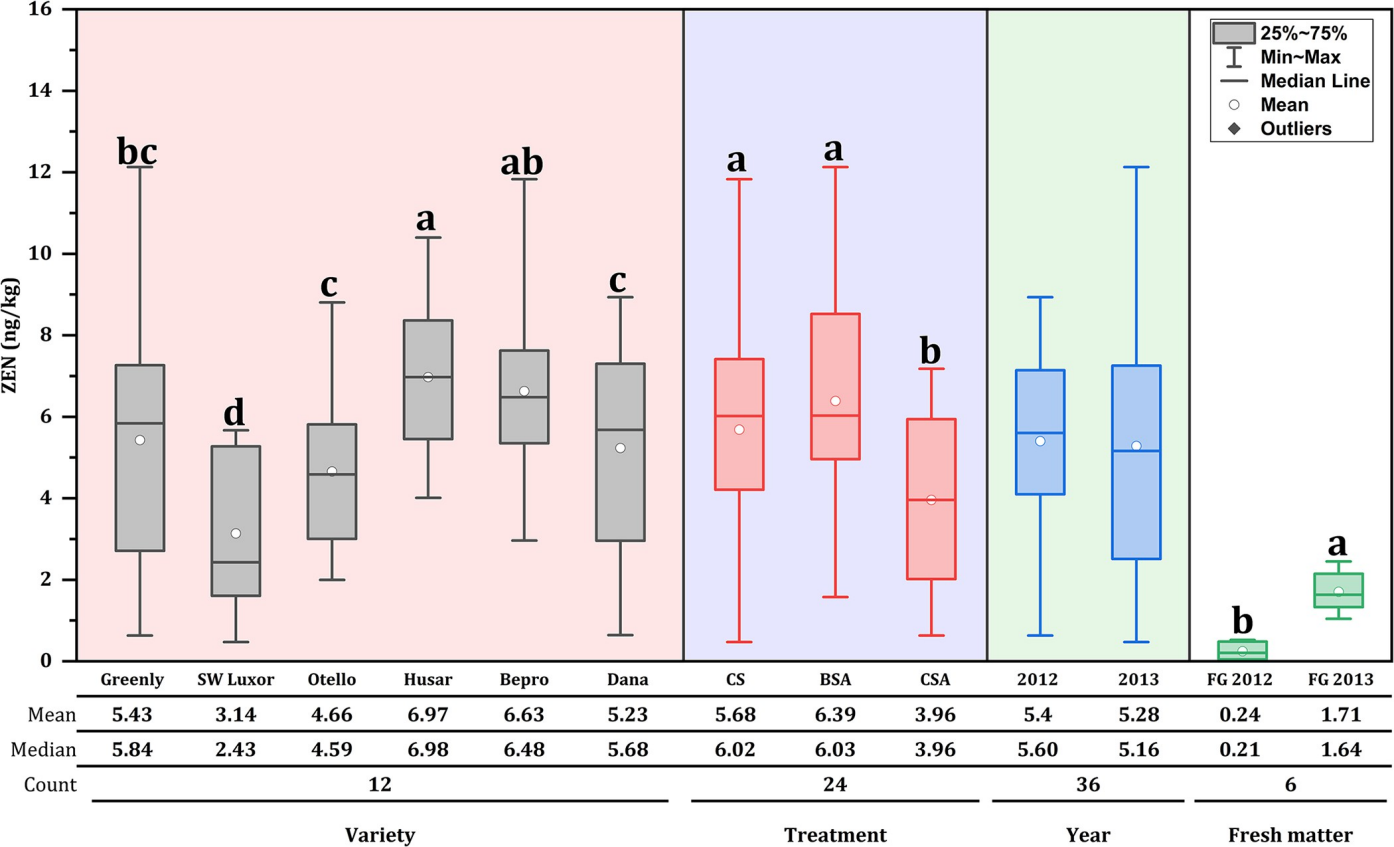
ZEN concentration (ng/kg DM) in six ensiled orchardgrass varieties and fresh matter. CS: Control silage; BSA: Biological silage additives; CSA: Chemical silage additives; FG: Fresh grass.

In general, in **Figs 1** and **2**, DON and ZEN concentration varied depending on ensiled varieties, additives, years and fresh matter.

Regarding silage grass, no significant difference (*p* > 0.05) was found between all orchardgrass varieties for DON concentration. As regards additives, the application of biological inoculants severely reduces (*p* < 0.05) DON concentrations compared to untreated silage group. Conversely, silage treated with chemical additives significantly decreased (*p* < 0.05) ZEN concentration compared to untreated silage group. Particularly, Husar variety exhibited a higher (*p* < 0.05) ZEN concentration compared to Sw-Luxor, Otello and Dana varieties.

In terms of years, 2013 showed a higher (*p* < 0.05) DON concentration compared to 2012. While for ZEN concentration, no significant differences (*p* > 0.05) were found in both years (2012 and 2013). In addition, in terms of fresh matter, no significant differences (*p* > 0.05) were found for DON concentration between years; however, ZEN concentration was higher (*p* < 0.05) in 2013 than in 2012.

**Fig 3:**
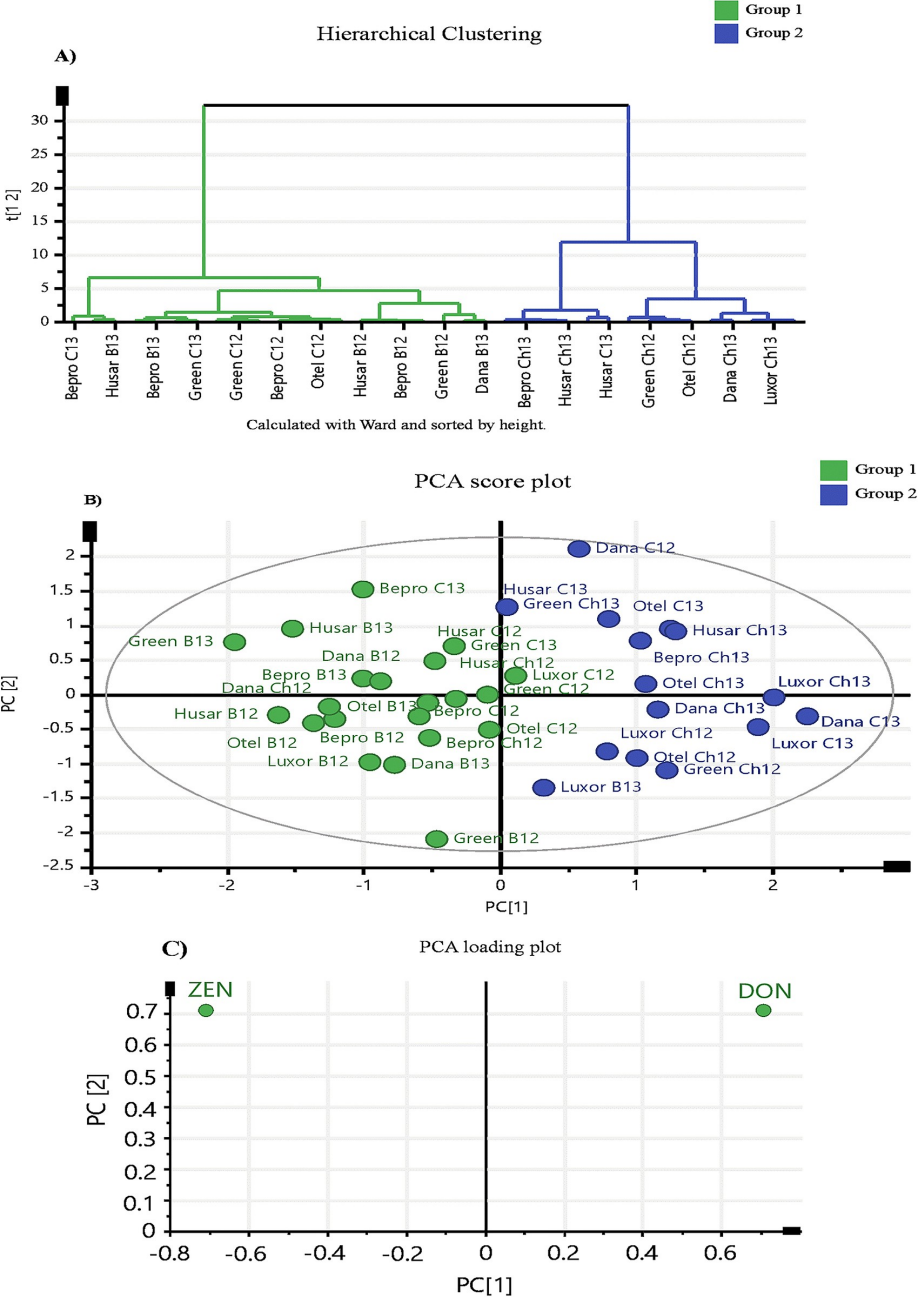
(A) Dendrogram of total DON and ZEN mycotoxin contents obtained by HCA shorted by the heigh using the ward method. (B) Score plot obtained from a PCA of the different orchardgrass varieties treated with additives correlated with DON and ZEN concentrations. (C) Loading plot obtained from a PCA.

With the aim of obtaining a better differentiation among treated silages as well as an evaluation of the most adequate additive for the prevention of mycotoxin contamination for each silage variety, a multivariate analysis was carried out. Two clusters were formed according to similarities among mycotoxin contents in orchardgrass varieties using a Hierarchical Cluster Analysis (HCA). As can be seen in **Fig 3A**, each cluster grouped a total of 9 samples (distinguished by green and blue colors). Similarly, to HCA, PCA (principal component analysis) provides the selection of the most important variables and the grouping of samples based to their mycotoxin contents (the same colors were employed for HCA and PCA) (see **Fig 3B**). Two groups were reported like HCA analysis where the samples from the same group contain similar mycotoxin content. Upon examining the overlap between **Fig 3B and 3C**, it was observed that green samples (group 1) contained the lowest DON concentration while blue samples situated in the opposite quadrant (group 2) had the highest DON concentration according to the PCA loading plot. In general, this analysis reflected that biological additives were the most effective for preventing DON contamination (group 1), whereas the chemical additive was more efficient for preventing ZEN contamination (group 2). Particularly, differences were observed in varieties such as Husar, Bepro, and Dana from 2012 (group 1), as well as for Sw-Luxor and Otello from 2012 and Greenly from 2013 (group 2) where chemical additive showed a positive prevention of DON contamination than biological additive. Moreover, the biological additive had a positive influence on the prevention of ZEN contamination in Sw-Luxor in 2013 than chemical additive.

## Discussion

Fungal spoilage is associated with mycotoxin production in silages and is the main concern in animal nutrition because leads to nutrient, dry matter losses and deteriorates the organoleptic properties of silage [30]. *Fusarium* is one of the most widespread mycotoxigenic species in crops and a primary contributor to mycotoxin contamination in animal feeds [31].

In light of the potential risk of mycotoxin contamination, we consider it important to investigate the combination of two relevant fungal metabolites DON and ZEN, in orchardgrass silage.

To avoid the health risks posed by these mycotoxins, it is necessary to find strategies to prevent and control mycotoxin production in silage. One applicable method to suppress or reduce the bioavailability of mycotoxins in animal diets is the use of bacterial inoculants [7]. Because fermentative bacteria have demonstrated the ability to mitigate the growth of molds, by acting as biological detoxifiers of ensiled forages, thus decreasing mycotoxin production in silage [16, 17, 32–34].

Additionally, certain mold-inhibiting chemical additives can help also reduce the contamination levels of dairy cow feeds. Recent studies conducted in Slovakia (with maize silage) and Finland (with grass silage) demonstrated that chemical additives such as urea and formic acid reduce ZEN concentration, but does not limit the increase of DON [20, 21].

Our results visibly proved that the presence of mycotoxins in the control silage allows clear differences to be made about the effects and the interaction of silage additives on mycotoxins **Figs 1 and 2**. Regarding varieties, Dana and Husar showed a higher concentration of mycotoxins in the silage with values of 23.99 ng/kg for DON and 6.97 ng/kg for ZEN, respectively, being the varieties most susceptible to mycotoxin contamination compared with the rest of the varieties. These findings are consistent with the study conducted by Skládanka et al. [35], where the concentration of mycotoxins in grass silage of *Festulolium pabulare* and *Lolium perenne* treated with additives (biological and chemical) denoted a higher content of DON at 156.73 μg/kg and ZEN at 66.07 μg/kg, respectively.

Moreover, our results revealed that silage from orchardgrass contained mycotoxins originating from *Fusarium* in both years (2012 and 2013) indicating the products of both pre- and post-ensiled molds were present. Although silages had different concentration profiles and ranges across both years (2012 and 2013), there was an increase in the incidence of mycotoxin detection in the year 2013 for DON while for ZEN no differences were observed. Our results differ from those presented by Skládanka et al. [35] in grass silage, who showed a three-year study (2008, 2009, and 2010) with high DON concentrations at 164.61, 156.49 and 141.73 μg/kg, respectively, with no statistical significance among years, while for ZEN the concentrations were 53.95, 73.24 and 41.81 μg/kg, respectively, with significant differences among years. It is evident that both fusariotoxins (DON and ZEN) concentration in ensiled grasses was affected by the year, with higher mycotoxin levels observed in 2008 and 2009 compared to 2010. This could be attributed to the fact that the temperature in the Central European region (Czech Republic) was lower in the years (2008 and 2009) by 0.3 and 0.7°C, respectively, compared to 2010.

In this context, our results of both fusariotoxins in silage grass (**Tables 2** and **3**) are below the threshold stipulated by the European Commission, where the concentration of DON range from ~ 21.86 to 37.26 ng/kg, ~10.21 to 15 ng/kg, ~20.72 to 29.14 ng/kg; and ZEN range from ~3.42 to 7.87 ng/kg, ~3.85 to 8.62 ng/kg and ~2.15 to 5.08 ng/kg, in control, biological and chemical silages, respectively. This suggests that the incidence of mycotoxins was too low in the current study compared to studies in maize silage conducted in Poland [36], Belgium [37], England [38], and Spain [39], where the maximum values of mycotoxin concentration found were 7.860, 8.912, 7.111 and 6.685 mg/kg for DON and 0.696, 3.124, 3.901 and 0.820 mg/kg for ZEN, respectively.

Moreover, the obtained results in our study differed from those published by Cogan et al. [38], where 51 samples of the grass silage were analyzed, and none of sample was tested positively for the presence of mycotoxins. However, our findings support the results published by Panasiuk et al. [1] and Dänicke et al. [40]. These authors determined in grass silage the maximum concentration of DON at 0.528 and 0.430 mg/kg; and ZEN at 0.009 and 0.080 mg/kg, respectively. In a new research study with grass silage, Dänicke et al. [41] assessed only DON at concentrations of 0.132 mg/kg and no value was detected for ZEN. Nonetheless, Penagos-Tabares et al. [42] have found a higher ZEN concentration in grass silage 0.668 mg/kg, which exceed the EU guidance level of 0.5 mg/kg (for ZEN in complete feedstuffs for ruminants), while DON concentration (29.6 μg/kg) was well below the established values.

Based on our study and other researchers’ findings, it is evident that the concentrations of ZEN and DON can vary from year to year. Factors such as climate change, specific regions, and the forage material used in the study directly impact the occurrence of *Fusarium* mycotoxins. Likewise, our results of DON and ZEN contamination in grass silage are in line with the aforementioned researchers’ findings. Furthermore, it is essential to thoroughly evaluate the occurrence of mycotoxins in grass silage, considering the synergistic relationships between individual mycotoxins.

In general, compared to the guidance values of EFSA [9, 10], our contamination levels of both fusariotoxins in grass silage can be regarded as low background contamination. This observation agrees with the studies realized in Austria and Poland, where the researchers found that grass silage from different farms was frequently contaminated with DON and ZEN, although at relatively low levels (e.g., 139 μg/kg for DON and 9.14 μg/kg for ZEN; 19.6 μg/kg for DON and 178 μg/kg for ZEN, respectively.) [1, 42]. In addition, a study conducted by Skládanka et al. [35] in the Czech Republic using biological and chemical additives also revealed low concentrations of DON and ZEN in grass silage of *Lolium perenne*, *Festulolium pabulare*, *Festulolium braunii*, and mixtures of these species with *Festuca rubra* or *Poa pratensis* (141.39, 156.73, 143.6, 161.97 and 167.7 μg/kg for DON and 66.07, 47.92, 46.34, 66.89, and 54.46 μg/kg for ZEN, respectively). Consequently, it is necessary to emphasize that in three studies (Austrian, Polish, and Czech) the concentrations of DON and ZEN-regulated toxins were considerably lower than the guideline values recommended by the European Commission. In addition, Panasiuk et al. [1] and Dänicke et al. [41] confirmed that the occurrence of DON and ZEN in maize silage is higher than in grass silage. This is likely a result of the fact that fungal organisms can easily survive on maize crops, which are richer in necessary proteins and polysaccharides than forage grass [43].

The production of feedstuffs such as silage in farm silos without any mycotoxins is very difficult as certain factors that promote mycotoxin production are out of human control [6].

The current study demonstrated that DON concentration was higher than ZEN in all ensiled orchardgrass varieties. It should be noted that DON and ZEN concentrations depended on the orchardgrass variety taking into account that all orchardgrass varieties were submitted to the same experimental conditions during pre- and post-ensiling. In this sense, higher contamination by mycotoxins was observed in 2013 than in 2012, for DON while for ZEN no statistical differences were observed.

This fact could be attributed to meteorological conditions during vegetation period, as the measured precipitation and temperature values in 2013 were higher by 47 mm and 0.5°C, respectively, compared to those in 2012. In this way, the climate change impacts on bioclimatic conditions, characterized by the co-occurrence of co-varying environmental variables, often affects the plant epiphytic microbial community occurrence differently. Thus, the orchardgrass varieties belonging to this study were raised in the Czech Republic, which is a country that the climate of the temperate climate zone presents which facilitate the growth of mycotoxins and, in particular, of DON mycotoxin. While ZEN mycotoxin growth is favored by warm and temperate climates [44].

As regards additives, the silage treated with biological and chemical additives had a positive effect on mycotoxin production by decreasing and counteracting the production of DON and ZEN, respectively, compared to control silage. In that way, the present study demonstrated that both additives had a reductive spectrum in the production of mycotoxins, each of them being very specific for each mycotoxin. On the other hand, our findings differed from the results reported by Skládanka et al. [35], who evinced that the biological–enzymatic inoculant did not reduce the concentration of DON, while the use of chemical additives drastically increased the content of DON compared to control silage. Likewise, in his study, they revealed that both additives (biological and chemical) reduced the concentration of ZEN, but with no statistical differences compared to untreated silage. On the other hand, Juráček et al. [45], denoted that the use of biological additives had a negative impact on rye silage by increasing DON and ZEN concentrations with significant differences compared to control silage; however, our findings are not consistent with this statement. Furthermore, our findings are partially in line with the study conducted by Franco et al. [21] in grass silage. They showed that the chemical additive reduced ZEN concentration compared to control silage but differ from our results when the chemical additive causes an opposite effect in DON concentration.

In fact, compared with our study, this proves that the matrix and the type of additive used for ensiling greatly influence the proliferation of mycotoxins in grass silage.

The common co-occurrence of DON and ZEN in silage makes it a critical issue for the agri-food industry, with a fundamental understanding required to develop mitigation strategies. The incidence of fusariotoxins in feeds and silages can be a potential threat to ruminants, due to the inadequate biodegradation of mycotoxins by rumen microflora that can negatively affect the productive and reproductive system of animals. However, to date, information on the effect of additives on the occurrence of mycotoxins in grass silage is scarce, especially for *Fusarium* toxins. Therefore, additional data on the toxicity in ruminants of DON and ZEN mycotoxins are needed to establish safe limits specifically for silage.

## Conclusion

The study of additives as reducing or neutralizing agents of mycotoxigenic fungal contamination in grass silages, particularly, has not been extensively explored to date.

In this study, our results demonstrate the efficacy of both biological and chemical additives in counteracting DON and ZEN contamination in different varieties of *Dactylis glomerata* L. silage for the first time. In general, biological additives prevented the proliferation of DON mycotoxin while chemical additive prevented the proliferation of ZEN mycotoxin.

Notably, the varieties Husar, Bepro, and Dana from 2012 (group 1), as well as Sw-Luxor and Otello from 2012 and Greenly from 2013 (group 2) treated with chemical additive showed a positive effect on the prevention of DON rather than ZEN. Likewise, the biological additive had a positive impact by preventing ZEN contamination rather DON for Sw-Luxor variety from 2013.

In addition, it should be noted that mycotoxin levels of both fusariotoxins (DON and ZEN) after the treatment with biological and chemical additives were below the thresholds imposed by the EU; nevertheless, the efficacy of both additives on mycotoxin production in orchardgrass silage was corroborated by reducing its content compared to the control silage. Therefore, fostering international awareness and knowledge about the role mycotoxins can play in silage hygienic safety is of key importance. Consequently, this collective understanding aims to prevent disorders in both animal and human health, emphasizing the need to establish safe limits specifically for silage.
